# Moss genetics and the way forward

**DOI:** 10.1371/journal.pgen.1007330

**Published:** 2018-05-10

**Authors:** Neil Ashton

**Affiliations:** Department of Chemistry and Biochemistry, University of Regina, Regina, Saskatchewan, Canada; University of California, Riverside, UNITED STATES

The moss *Physcomitrella patens* was originally chosen for the study of gene regulation in plant development [**1**]. Its gametophyte generation is haploid and exhibits an ordered sequence of developmental transitions facilitating the isolation and identification of mutants. The physiology associated with this sequence had been partially characterised in the closely related *Funaria hygrometrica* and, for example, shown to involve auxin [**2**] and cytokinin [**3**]. *P*. *patens* was preferred to *F*. *hygrometrica* because of the invaluable preliminary studies of Engel [**4**] and the short, approximately 8-week life cycle of *P*. *patens* in laboratory conditions. Consequently, pioneers of the *P*. *patens* plant model system envisaged using classical forward genetics for the dissection of developmental processes. Since protonemal tissue, the first tissue to be formed from a germinating moss spore, can be propagated vegetatively, the culture of mutants that were abnormal even at an early stage of development presented no problems—although when such mutants were sterile, orthodox genetic analysis was not possible. To circumvent this problem, somatic hybrids were made by protoplast fusion [**5**]. Perhaps the most complete early example of this forward genetics approach involved 15 sterile gametophore overproducing (*ove*) mutants [**6**]. Somatic hybridisation demonstrated that the ove phenotype is recessive in all but one case, and the ratio of mutant to wild-type postmeiotic segregants from self-fertilised hybrids was consistent with the altered phenotype being the result of a single mutation in each case tested. Recessive mutants were assigned to three complementation groups (*ove*A, B, and C) and the ratio of *ove* to wild-type segregants from a selfed *ove*A/*ove*B somatic hybrid showed that the two affected genes are unlinked [**7**]. The biochemical basis for the ove phenotype is an increased level of biologically active cytokinins, principally N^6^-(Δ^2^-isopentenyl) adenine [**8, 9**]. However, mapping the *ove* mutations and identifying the genes affected and their corresponding proteins have yet to be achieved.

Forward genetic dissection of a developmental process using somatic hybridisation is compromised when the causal mutations are dominant [**10**]. Thus, it was another three decades before a candidate-gene strategy showed that 8 of 17 chemically-induced *nar* (1-naphthalene acetic acid and indole-3-acetic acid resistant) strains harbour mutations distributed among the three *P*. *patens* homologues of *Arabidopsis Aux*/*IAA* (auxin/indole-3-acetic acid) genes [**11**].

Consequently, forward genetic analysis in *P*. *patens* was unattractive to most researchers, and their disaffection was exacerbated by the paucity of markers required for effective mapping of new mutations that existed prior to the generation of a genetic linkage map based on numerous molecular markers and anchored to the genome sequence [**12**]. By contrast, the exceptionally high rate of homologous recombination in *P*. *patens* [**13]**, comparable to that in *Saccharomyces cerevisiae*, and the availability of its complete genome sequence **[14**] led to an explosion of reverse genetics functional studies that exploited these advantages.

Recently, however, the speed, accuracy, and modest cost of next-generation DNA sequencing (NGS) have provided a catalyst for mapping and identifying mutations in mutant plants generated by a forward genetics approach. One of the first successful *P*. *patens* studies utilised comparative whole-genome sequencing to identify the gene affected in AR7, a UV-induced mutant with reduced abscisic acid (ABA) sensitivity and reduced tolerance to hyperosmosis. The sequenced genome of AR7 was analysed against the reference genome sequence of *P*. *patens*, revealing 2,068 mutations in AR7 that caused 47 nonsynonymous substitutions in encoded proteins. To identify the mutated gene responsible for the AR7 phenotype, cDNAs from the 47 candidate genes were tested individually for their ability to rescue the mutant phenotype. Only one cDNA was effective. The corresponding gene, designated *ARK* (ABA and abiotic stress-responsive Raf-like kinase), encodes a B3 Raf-like mitogen-activated protein (MAP) kinase kinase kinase involved in integrating ABA and osmotic signals upstream from sucrose nonfermenting 1 (SNF1)-related protein kinase 2, known to be a central regulator of stress signaling in plants [**15**]. Although this is an impressive example of forward genetics and gene identification, the overall approach is perhaps too time-consuming for general use. Two recent studies have employed more economical approaches based on the next-generation mapping (NGM) strategy proposed and tested with *Arabidopsis* genes by Austin and colleagues [**16]**. One of them [**17**] independently mapped and identified an ABA nonresponsive (*ANR*) gene, which, it transpires, is the *ARK* gene.

**Fig 1:**
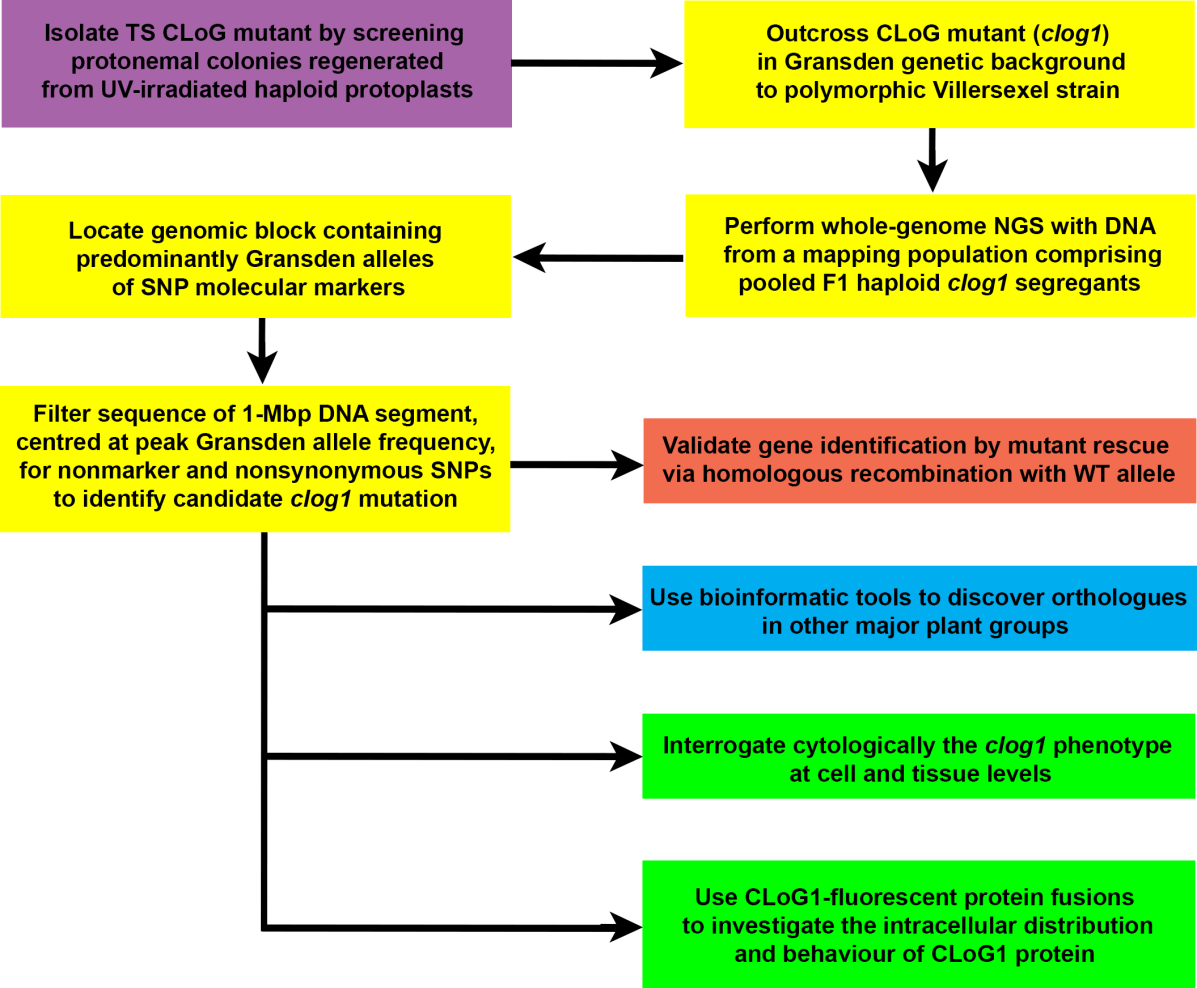
Outline of the major elements of forward genetics as envisaged by Ding and colleagues. Proof of concept was achieved by mapping and characterising *clog1*. Coloured panels indicate different aspects of the study as follows: Violet panel—UV mutagenesis of protoplasts and screening for TS CLoG mutants. Yellow panels—NGM of *clog1* mutation. Red panel—Validation of *clog1*. Blue panel—Bioinformatic search for *CLoG1* orthologues in other plant groups. Green panels—Phenotypic interrogation of *clog1*. CLoG, conditional-loss-of-growth; NGM, next-generation mapping; NGS, next-generation sequencing; TS, temperature-sensitive; WT, wild-type.

The other study (Fig 1), undertaken by Ding and colleagues [**18**] and reported in the May 2018 issue of *PLOS Genetics*, used temperature-sensitive (TS) growth mutants, which were blocked at an early stage of gametophytic development and were sterile at the restrictive temperature (32°C) but grew normally and were fertile at the permissive temperature (25°C). Although a few TS mutants of *P*. *patens* have been studied to discern the biochemical and metabolic basis of their altered phenotypes, this is the first case in which such a mutant has been used to overcome the problem of sterility associated with some mutant phenotypes, thereby allowing forward genetic analysis to be performed via sexual crossing. The authors used NGM to map the causal mutation and identify the affected gene in *clog1*, a UV-induced CLoG (conditional-loss-of-growth) mutant. They generated a mapping population of haploid F1 plants by outcrossing *clog1*, which possesses a Gransden genetic background, to a polymorphic Villersexel (Vx) strain, which expresses mCherry (Vx::mCherry). This enabled facile identification at 25°C, the permissive temperature, of crossed sporophytes by their mCherry fluorescence on nonfluorescent gametophores of *clog1* [**19**]. Twenty-four TS mutant segregants were selected from protonemal progeny grown from haploid meiospores produced by the hybrid sporophytes, and their pooled DNA was used for whole-genome NGS. The key to NGM in this case was recognising that the *clog1* mutation would be located within a genomic block containing predominantly Gransden molecular markers with allele frequencies tending to a peak value of 1. Contrastingly, the rest of the pooled *clog1* segregants’ genome would consist of an equal mixture of DNA derived from both parental strains with neutral marker allele frequencies of approximately 0.5 as a result of random crossing-over during meiosis. Only one such genomic block was discovered and mapped to a position approximately 4.6 Mbp from the left end of chromosome 24. The sequence of a 1-Mbp DNA segment centred at the peak Gransden allele frequency was filtered for nonmarker and nonsynonymous SNPs, thereby identifying a single candidate mutation for *clog1*. Its identity was validated by rescuing the conditional phenotype by homologous recombination with the wild-type allele. Bioinformatic analysis of *CLoG1* revealed that it encodes a previously uncharacterised protein that is present in plants belonging to all the major groups from algae to angiosperms. Fluorescent protein fusions of CLoG1 indicated that it is localised to microtubules and displays an unusual behaviour, appearing to track both ends of depolymerising microtubules. *Clog1* possesses smaller cells than wild type but can complete cell division, suggesting that functional CLoG1 is necessary for cell growth but is not essential for cell division.

Ding and colleagues have impressively demonstrated that forward genetics approaches in *P*. *patens* now have the power to identify novel genetic functions and the genes responsible for them without a priori knowledge. Nevertheless, the full potential of forward genetics as a means of interrogating the genetic complexity underlying a chosen phenotype that is dependent upon the activities of several or many genes remains unrealised. That said, Ding and colleagues have convincingly shown us the way forward, and, for example, NGM of mutations in strains representing the three *ove* complementation groups discussed towards the start of this perspective might at last identify the affected genes four decades after *ove* mutants were discovered.
