# Correction: Moss genetics and the way forward

**DOI:** 10.1371/journal.pgen.1007446

**Published:** 2018-06-07

**Authors:** The PLOS Genetics staff

There is an error in the URL associated with reference 18. The correct reference is: Ding X, Pervere LM, Bascom Jr. C, Bibeau JP, Khurana S, Butt AM, et al. Conditional genetic screen in *Physcomitrella patens* reveals a novel microtubule depolymerizing-end-tracking protein. PLoS Genet. 2018; 14(5): e1007221. https://doi.org/10.1371/journal.pgen.1007221. The publisher apologizes for the error.
